# Supplementary material for: Matching sensor ontologies through siamese neural networks without using reference alignment
Source: PeerJ Comput Sci. 2021 Jun 18;7:e602. doi: 10.7717/peerj-cs.602 (PMC8237319; doi:10.7717/peerj-cs.602)
Supplement: Supplemental Information 1 [file peerj-cs-07-602-s001.zip › 261/onto.html]

Author: Nick Knouf <nknouf@mit.edu>  
Contributor: Antoine Zimmermann <antoine.zimmermann@inrialpes.fr>, Jérôme Euzenat,   
Date: 08/06/2005  
Version: $Id: onto-exp.rdf,v 1.10 2008/05/27 14:40:02 euzenat Exp $

## Classes

**http://www.w3.org/1999/02/22-rdf-syntax-ns#List** (, *)*


**http://xmlns.com/foaf/0.1/Person** (, *)*


**http://xmlns.com/foaf/0.1/Organization** (, *)*


**uqwz** (, *)*
:   **sqdsq** (, *)*
    :   **PBdgcs** (, *)*
        :   **zajdhqs** (, *)*
            :   **dzajndsq** (, *)*


                **sqxsqkd** (, *)*


                **zdqssqdb** (, *)*

            **qsdsnbsqd** (, *)*
            :   **zdzaudbd** (, *)*


                **scds** (, *)*


                **dcsqdsq** (, *)*
                :   **ZikPzsz** (, *)*


                    **ZGKW1E** (, *)*

                **zeadza** (, *)*

            **sqdsopq** (, *)*
            :   **RJbGVM** (, *)*
                :   **hazdn** (, *)*


                    **Be** (, *)*


                    **fGfFm** (, *)*


                    **wFuU** (, *)*

                **dzqndbzq** (, *)*


                **vccfsq** (, *)*


                **dcsqdcsqd** (, *)*


                **deqdxcsqcsq** (, *)*

            **zdazsx** (, *)*
            :   **xsqlknk** (, *)*


                **zqdzqzai** (, *)*

            **wxsdxqsdh** (, *)*


            **dqzdxdcsqj** (, *)*
            :   **EwQu** (, *)*
                :   **xhNJuGU** (, *)*


                    **UWmlX** (, *)*


                    **FPVijs** (, *)*
                    :   **cnNCBO** (, *)*
                        :   **ZVxsNo** (, *)*


                            **jZVxsNo** (, *)*

                        **Lluuk** (, *)*
                        :   **sdcsqhyz** (, *)*


                            **QMgwFB** (, *)*


                            **YeLgZJu** (, *)*


                            **LXtNU** (, *)*

                    **XaWa** (, *)*
                    :   **FkmaDDV** (, *)*

                **azzauhuie** (, *)*
                :   **pjDVyIr** (, *)*

        **dscdscg** (, *)*

**BeXCIG** (, *)*
:   **qsdsquj** (, *)*


    **wxVRZcIXbbz** (, *)*

**cexQ** (, *)*
:   **zqedzbx** (, *)*


    **RcrfZK** (, *)*


    **qHdl** (, *)*


    **izVyrw** (, *)*

**qzddj** (, *)*


**izxnquo** (, *)*
:   super: *http://xmlns.com/foaf/0.1/Organization*  


    **YpNk** (, *)*


    **zauio** (, *)*


    **BriFsw** (, *)*
    :   **MyRFOvC** (, *)*


        **zadazxn** (, *)*


        **SNNu** (, *)*


        **SLoVq** (, *)*

**dsqdbz** (, *)*
:   super: *http://www.w3.org/1999/02/22-rdf-syntax-ns#List*

**dnqsb** (, *)*


**dzshyuua** (, *)*

## Properties

**http://www.w3.org/1999/02/22-rdf-syntax-ns#first**: http://www.w3.org/1999/02/22-rdf-syntax-ns#List -> \_ *()*


**http://www.w3.org/1999/02/22-rdf-syntax-ns#rest**: http://www.w3.org/1999/02/22-rdf-syntax-ns#List -> http://www.w3.org/1999/02/22-rdf-syntax-ns#List *()*

**http://purl.org/dc/elements/1.1/creator**\_ -> \_ *()*


**http://purl.org/dc/elements/1.1/contributor**\_ -> \_ *()*


**http://purl.org/dc/elements/1.1/description**\_ -> \_ *()*


**http://purl.org/dc/elements/1.1/date**\_ -> \_ *()*


**http://xmlns.com/foaf/0.1/firstName**\_ -> \_ *()*


**#lastName**\_ -> \_ *()*


**http://xmlns.com/foaf/0.1/name**\_ -> \_ *()*

## Individuals

<rdf:List@ttp://www.w3.org/1999/02/22-rdf-syntax-ns#nil>


<foaf:Person@a04570373>
:   - foaf:name = 'John-Jules Meyer'
    - foaf:firstName = 'John-Jules'

<foaf:Person@a43836633>
:   - foaf:name = 'Jeen Broekstra'
    - foaf:firstName = 'Jeen'

<foaf:Person@a85228505>
:   - foaf:name = 'Alexander Mädche'
    - foaf:firstName = 'Alexander'

<foaf:Person@a48552212>
:   - foaf:name = 'Björn Schnizler'
    - foaf:firstName = 'Björn'

<foaf:Person@a971541439>
:   - foaf:name = 'Alberto Trombetta'
    - foaf:firstName = 'Alberto'

<foaf:Person@a11090777>
:   - foaf:name = 'Christine Parent'
    - foaf:firstName = 'Christine'

<foaf:Person@a250331360>
:   - foaf:name = 'R. Schmidt'
    - foaf:firstName = 'R.'

<foaf:Person@a79573306>
:   - foaf:name = 'York Sure'
    - foaf:firstName = 'York'

<foaf:Person@a885257047>
:   - foaf:name = 'M. Punceva'
    - foaf:firstName = 'M.'

<foaf:Person@a74993404>
:   - foaf:name = 'I. V. Levenshtein'
    - foaf:firstName = 'I. V.'

<foaf:Person@a71003986>
:   - foaf:name = 'Steffen Staab'
    - foaf:firstName = 'Steffen'

<foaf:Person@a572406328>
:   - foaf:name = 'Frank Boer'
    - foaf:firstName = 'Frank'

<foaf:Person@a139477786>
:   - foaf:name = 'Maarten Menken'
    - foaf:firstName = 'Maarten'

<foaf:Person@a337716610>
:   - foaf:name = 'Manfred Hauswirth'
    - foaf:firstName = 'Manfred'

<foaf:Person@a086379337>
:   - foaf:name = 'Wiebe Hoek'
    - foaf:firstName = 'Wiebe'

<foaf:Person@a712561038>
:   - foaf:name = 'Marc Ehrig'
    - foaf:firstName = 'Marc'

<foaf:Person@a066600210>
:   - foaf:name = 'Danilo Montesi'
    - foaf:firstName = 'Danilo'

<foaf:Person@a093016135>
:   - foaf:name = 'Rogier Eijk'
    - foaf:firstName = 'Rogier'

<foaf:Person@a944339054>
:   - foaf:name = 'Frank van Harmelen'
    - foaf:firstName = 'Frank'

<foaf:Person@a98078619>
:   - foaf:name = 'Philippe Cudré-Mauroux'
    - foaf:firstName = 'Philippe'

<foaf:Person@a39510672>
:   - foaf:name = 'Z. Despotovic'
    - foaf:firstName = 'Z.'

<foaf:Person@a431956276>
:   - foaf:name = 'Stefano Spaccapietra'
    - foaf:firstName = 'Stefano'

<foaf:Person@a431956276b>
:   - foaf:name = 'Mike Papazoglou'
    - foaf:firstName = 'Mike'

<foaf:Person@a431956276c>
:   - foaf:name = 'Zahir Tari'
    - foaf:firstName = 'Zahir'

<foaf:Person@a70955601>
:   - foaf:name = 'A. Datta'
    - foaf:firstName = 'A.'

<foaf:Person@a467748807>
:   - foaf:name = 'Ateret Anaby-Tavor'
    - foaf:firstName = 'Ateret'

<foaf:Person@a3105947>
:   - foaf:name = 'Ronny Siebes'
    - foaf:firstName = 'Ronny'

<foaf:Person@a29105611>
:   - foaf:name = 'Karl Aberer'
    - foaf:firstName = 'Karl'

<foaf:Person@a958684218>
:   - foaf:name = 'Peter Mika'
    - foaf:firstName = 'Peter'

<foaf:Person@a94533498>
:   - foaf:name = 'Peter Haase'
    - foaf:firstName = 'Peter'

<foaf:Person@a900366022>
:   - foaf:name = 'Avigdor Gal'
    - foaf:firstName = 'Avigdor'

<qsdsquj@a246119474>
:   - foaf:name = 'Journal of Web Semantics'

<zauio@a131020767>


<qsdsquj@a70981683>


<zauio@a85849488>


<qsdsquj@a362042121>


<qsdsquj@a674639524>


<qsdsquj@a906774044>


<izVyrw@spg04>


<zqedzbx@a72192307c>


<zqedzbx@a32071928c>


<zdqssqdb@a060097576>


<deqdxcsqcsq@a64263824>


<deqdxcsqcsq@a439508789>


<hazdn@a492378321>


<hazdn@a475526642>


<vccfsq@a71568377>


<zdqssqdb@a72192307>


<zdqssqdb@a32071928>


<wxsdxqsdh@a140583454>


<deqdxcsqcsq@a11065952>


<hazdn@a80299267>


<dzajndsq@a108048723>


<hazdn@a456080390>


<hazdn@a846015923>

---

Generated by OWL2HTML
